# Development of KASP molecular markers and construction of fingerprinting for cowpea (*Vigna unguiculata* (L.) Walp.) based on ddRAD-Seq

**DOI:** 10.1371/journal.pone.0343756

**Published:** 2026-08-14

**Authors:** Juan Xiang, Zhuoling Zhong, Chengming Zhang, Min He, Kun Cai, Lanping Gu, Li Xu, Shilin Su, Yi Zou, Jie Li, Kehao Cui, Huimin Qiu, Bengang Xian, Shaohong Fu, Ling Chen, Xiaowei Liu

**Affiliations:** 1 Chengdu Academy of Agricultural and Forestry Sciences, Chengdu, China; 2 Chengdu Agricultural College, Chengdu, China; 3 College of Chemistry and Life Sciences, Sichuan Provincial Key Laboratorymfor Development and Utilization of Characteristic Horticultural Biological Resources, Chengdu Normal University, Chengdu, China; Louisiana State University College of Agriculture, UNITED STATES OF AMERICA

## Abstract

Cowpea (*Vigna unguiculata* (L.) Walp.) is a globally important legume crop. However, the scarcity of efficient molecular markers has hindered molecular breeding efforts and the protection of plant breeders’ rights. In this study, we employed double-digest restriction-site associated DNA sequencing (ddRAD-seq) to characterize the genetic diversity of 19 cowpea accessions. A total of 791,621 SNPs were identified, of which 13,469 high-quality SNPs were retained after filtering. Population structure and phylogenetic analyses revealed that these accessions clustered into three distinct groups. To facilitate cost-effective and rapid genotyping, we developed a panel of KASP (Kompetitive Allele-Specific PCR) markers. Through rigorous screening for polymorphism and stability, we identified six core KASP markers located in exonic regions. These six markers alone were sufficient to discriminate all 19 accessions. Based on these core markers, we constructed a unique DNA fingerprinting profile and assigned specific QR codes for each accession. This study demonstrates that selecting core KASP markers from ddRAD-seq data is a powerful strategy for germplasm identification. The developed fingerprinting system provides a robust, low-cost tool for seed purity testing, variety authentication, and marker-assisted selection in cowpea breeding programs.

## 1. Introduction

Cowpea (*Vigna unguiculata* (L.) Walp.) is an annual legume crop with high protein and carbohydrates contents, and it is also rich in a variety of vitamins and minerals, contributing to antioxidant capacity, anti-aging effects, and the maintenance of normal physiological functions [1]. Cowpea is commonly consumed as a vegetable in Asia, whereas in Africa it is mainly used for grains [2]. Cowpea is a diploid crop with a chromosome number of 2n = 2x = 22 and a genome size of approximately 620 Mb [3]. Continuous artificial selection during domestication and cultivation has driven genetic variation in multiple traits, resulting in diversified cultivar types [4,5]. Currently, most cowpea cultivars are selfed lines developed through conventional breeding and are highly similar phenotypically; thus, it is difficult to accurately distinguish cultivars by visual inspection, posing major challenges to traditional distinctness, uniformity, and stability (DUS) testing [6]. Therefore, to facilitate rapid cultivar registration while protecting breeders’ intellectual property, new technologies are urgently needed to support cowpea breeding and variety protection.

Molecular marker technologies are reliable tools for germplasm characterization, cultivar identification, and diversity analysis. Single nucleotide polymorphism (SNP) markers, regarded as third-generation molecular markers, are DNA sequence polymorphisms caused by single-nucleotide variation at the genome level. SNPs are among the most abundant DNA variants in plant genomes and are more likely to be associated with biological functions and phenotypes [7]. Because of their high genetic stability, broad distribution, and convenient detection, SNP markers have been widely used in crop diversity analysis, genetic map construction, association mapping, and marker-assisted selection [8,26]. Traditional SNP discovery requires substantial labor and time, whereas the development of next-generation sequencing (NGS) has greatly reduced the cost of whole-genome sequencing, promoted genotyping approaches that reduce genome complexity, and enabled the generation of large numbers of high-density SNPs as molecular markers [9]. Kompetitive allele-specific PCR (KASP) is a PCR-based fluorescent SNP genotyping technology developed in recent years. By using fluorescently labeled primers and fluorescence resonance energy transfer (FRET) cassettes to discriminate alleles, KASP offers low cost, high throughput, high accuracy, and high polymorphism. KASP has become one of the most widely used SNP genotyping platforms and marker detection methods, showing great potential for crop genotyping, fingerprinting, and germplasm identification [10–12].

DNA fingerprinting is an important application of molecular marker technology. By testing cultivars using DNA markers and establishing a profile based on genotypes at each marker,DNA fingerprinting can effectively avoid problems of “same name, different cultivar” and “different names, same cultivar” [13,14]. This approach has been recommended by the International Union for the Protection of New Varieties of Plants (UPOV) [15]. To date, fingerprinting systems based on KASP-derived SNP markers have been established in many crops, including sweetpotato [16], hemp [17], orchids [15], and cauliflower [18]. In cowpea, extensive efforts have been made to develop genomic resources to facilitate marker-assisted breeding. Muchero et al. developed an Illumina 1536-SNP GoldenGate genotyping array and applied it to 741 recombinant inbred lines from six mapping populations. Approximately 90% of the SNPs were technically successful, yielding 1,375 reliable markers, of which 928 were incorporated into a consensus genetic map [19]. Muñoz et al. [20], used resources developed from the African cultivar IT97K-499-35 and whole-genome sequencing (WGS) data from 36 additional cowpea populations to develop a 51,128 SNP genotyping assay and generate a consensus genetic map containing 37,372 SNPs. In this study, we used ddRAD-seq (double-digest restriction-site associated DNA sequencing) to identify polymorphic SNPs among 19 core cowpea accessions, developed KASP markers for genotyping, and constructed DNA fingerprints for these 19 accessions, providing a technical basis for marker-assisted breeding and cultivar identification in cowpea.

## 2. Materials and methods

### 2.1 Plant materials

The 19 cowpea accessions used in this study were obtained from the Institute of Horticulture, Chengdu Academy of Agriculture and Forestry Sciences. These 19 accessions were selected as a representative subset from 2,340 conserved cowpea accessions based on geographic origin, resource type, and phenotypic variation in key agronomic traits. The characteristics of the 19 accessions are shown in Table 1.

**Table 1:** Information on 19 cowpea accessions Simple.

ID	Other identifier	Resource Type	Origin	Leaf shape	Flower color	Commercial pod color	Pod shape	Seed color	Seed shape
170	Changlong No. 5	Commercial cultivar	Shandong, China	Long oval rhombus	White	Dark green	Long oval shape	Orange	Kidney-shaped
17FY	JD18	Breeding material	Sichuan, China	Lanceolate	Purple	Green	Long oval shape	Red	Kidney-shaped
18415	White rice bean	Local variety	Fujian, China	Oval	White	Dark green	Flat oval shape	White	Ellipse
18442	Black rice bean	Local variety	Henan, China	Oval	White	Green	Flat oval shape	Black	Ellipse
18443	Purple-grained rice bean	Local variety	Guangxi, China	Oval	Purple	Light green	Long oval shape	Purplish red	Rectangle
1858	Philippines 7	Commercial cultivar	Philippines	Oval	Purple	Light green	Flat oval shape	Red	Rectangle
19302-2-4	Yuliuxingwang	Commercial cultivar	Sichuan, China	Long oval rhombus	Purple	Green	Long oval shape	Orange-based brown flowers	Kidney-shaped
20351	Zhengyuan Green Cowpea	Commercial cultivar	Guangdong, China	Long oval rhombus	Purple	Dark green	Long oval shape	Black	Kidney-shaped
20479	S1120	Breeding material	Guangdong, China	Long oval rhombus	Purple	Pattern	Long oval shape	Red	Kidney-shaped
22565	Kafeixindilv	Local variety	Liaoning, China	Long oval rhombus	White flower	Dark green	Long oval shape	Red	Kidney-shaped
22572	DH28	Breeding material	Sichuan, China	Long oval rhombus	Purple	White pod	Long oval shape	Orange background with purple flowers	Kidney-shaped
22599	JD16	Breeding material	Jiangxi, China	Long oval rhombus	Purple	Green	Long oval shape	White	Kidney-shaped
JJ40-1-1	JD19	Breeding material	Sichuan, China	Long oval rhombus	Purple	Green	Long oval shape	Orange-based brown flowers	Kidney-shaped
jiang12	Chengjiang 12	Commercial cultivar	Sichuan, China	Long oval rhombus	Purple	Purplish red	Long oval shape	Red	Kidney-shaped
jiang14	Chengjiang 14	Commercial cultivar	Sichuan, China	Long oval rhombus	White	Dark green	Long oval shape	Orange	Kidney-shaped
jiang17	Chengjiang 17	Commercial cultivar	Sichuan, China	Long oval rhombus	Purple	Light green	Long oval shape	Red	Kidney-shaped
jiang30	Chengjiang 30	Commercial cultivar	Sichuan, China	Long oval rhombus	Purple	Green	Long oval shape	Orange-based brown flowers	Kidney-shaped
jiang6	Chengjiang 6	Commercial cultivar	Sichuan, China	Long oval rhombus	Purple	Dark green	Long oval shape	Red	Kidney-shaped
p-3	p-3	Breeding material	Sichuan, China	Long oval rhombus	Purple	Light green	Long oval shape	Black	Kidney-shaped

### 2.2 DNA extraction and re-sequencing

Cowpea seedlings were grown in plug trays. When the seedlings reached the three-leaf stage, young leaves from three biological replicates of each accession were collected for genomic DNA extraction using a modified CTAB method [21]. DNA quality was assessed by 1.0% agarose gel electrophoresis, and DNA concentration was measured using a NanoDrop 2000 UV spectrophotometer. The DNA samples were then diluted to 50 ng/μL.

For ddRAD-seq library construction, restriction enzymes were selected based on the availability of the cowpea reference genome. Briefly, the number and distribution of restriction sites for different enzymes were evaluated in silico using the reference genome sequence, and NlaIII (Hin1II, CATG^) and EcoRI (G^AATTC) were selected for double-digest library construction. Qualified DNA samples were used to construct paired-end ddRAD libraries. Restriction digestion and adapter ligation were performed sequentially. Briefly, 500 ng genomic DNA was first incubated with 0.6 U EcoRI (NEB), T4 DNA ligase (NEB), ATP (NEB), and EcoRI adapters containing sample-specific index sequences at 37°C for 3 h, followed by annealing at 65°C for 1 h. NlaIII (NEB) and NlaIII adapters were then added and incubated at 37°C for 3 h. After the reaction, the restriction enzymes were inactivated at 65°C for 30 min. Fragment size selection was performed by agarose gel electrophoresis, and DNA fragments of 400–600 bp were excised and recovered. The recovered DNA was quantified using Qubit 3.0 (Life Technologies), and 24 samples were pooled in equal amounts. Sequencing libraries were constructed using an Illumina TruSeq kit and sequenced by Genepioneer Biotechnologies (Nanjing, Jiangsu, China) on the Illumina NovaSeq 6000 platform with PE150 reads.

### 2.3 Raw sequencing data processing and SNP calling

Raw reads were subjected to quality control to remove low-quality reads and obtain clean reads. The clean reads were then aligned to the cowpea reference genome (NCBI accession: GCA_004118075.2) using BWA v0.7.19-r1273. SNP calling was performed using GATK v4.2.1.0 to obtain the initial set of SNPs. For downstream analyses, SNPs were further filtered based on mean sequencing depth, minor allele frequency (MAF), call rate, quality score, and biallelic status. Specifically, SNPs with a mean sequencing depth ≥ 5 × , MAF ≥ 0.05, SNP call rate per sample ≥ 0.7, quality score ≥ 30, and biallelic loci were retained for population structure analysis. The sequencing depth threshold (≥5×) was used to improve the reliability of genotype calling, while the call rate threshold (≥0.7) was applied to reduce the influence of excessive missing data while retaining sufficient informative loci for downstream analyses. All data processing steps were conducted using publicly available software or online analysis platforms.

### 2.4 PCA and population structure analysis

Principal component analysis (PCA) was performed using **GCTA** based on the filtered SNP dataset to infer sample clustering. Before PCA and ADMIXTURE analyses, linkage disequilibrium (LD) pruning was performed using PLINK with the parameter “--indep-pairwise 50 10 0.2” to remove highly linked SNPs and reduce marker redundancy. The resulting LD-pruned SNP dataset was then used for downstream population structure analyses. A phylogenetic tree was constructed using the maximum-likelihood method implemented in FastTree (v2.1.9). Population genetic structure was analyzed using ADMIXTURE based on the same LD-pruned SNP dataset.

### 2.5 Development of KASP primers

A total of 13,469 SNP markers with no other SNPs within 50 bp upstream or downstream were retained. After applying filters of mean depth ≥ 5 × , quality score > 30, minimum completeness > 0.9, minimum minor allele frequency > 0.05, and biallelic status, 11,490 SNP markers were obtained. For each of the 11,490 SNPs, 100 bp upstream and downstream flanking sequences were extracted and aligned to the reference genome using BLAST; markers mapping to multiple genomic positions were removed, yielding 6,958 SNP markers. Markers with polymorphism information content (PIC) > 0.35 were retained [22]. Primer3 (v2.4.0) was used to design primers for the 648 SNP markers obtained after the above filtering steps, and loci were further checked to avoid additional polymorphisms before conversion into KASP markers. The SNP markers selected in this study were primarily treated as neutral, high-confidence polymorphic markers for DNA fingerprint construction and germplasm identification. Their selection was based mainly on sequencing quality, polymorphism, genotyping stability, unique genomic mapping, and suitability for KASP assay development, rather than on known functional associations with specific genes or loci.

### 2.6 SNP Selection, construction of DNA fingerprint and KASP primer validation

Using these 19 cowpea accessions, the effectiveness of newly developed KASP primers was evaluated. Only loci that generated reliable genotyping results were retained as candidate loci. Data processing and filtering were performed using an online analysis platform provided by Genepioneer (http://cloud.genepioneer.com:9929/#/tool/alltool/detail/326), and six KASP primers that could discriminate the 19 accessions were ultimately selected, enabling construction of a DNA fingerprinting profile based on the core SNP markers. KASP genotyping was performed using DNA from three biological replicates per accession. For each primer set, standard FAM and HEX fluorescent tails were added to the 5′ ends of the two allele-specific forward primers. Primers were mixed at a ratio of forward primer 1: forward primer 2: common reverse primer = 2: 2: 5. Each 20 μL PCR reaction contained 2.8 μL primer mix, 2 μL DNA template, 10 μL master mix, and 5.2 μL ddH2O. PCR was performed in 96-well plates using a touchdown program from 61°C to 55°C: 95°C for 10 min; 10 touchdown cycles of 95°C for 15 s and 61°C for 45 s with the annealing temperature decreasing by 0.6°C per cycle; followed by 28 amplification cycles of 95°C for 15 s and 55°C for 45 s; and plate reading at 35°C for 30 s. Detailed information on the KASP-specific primers, including primer sequences, is provided in Supplementary S1 Table.

## 3. Results

### 3.1 ddRAD-seq analysis and SNP exploration

ddRAD-seq generated a total of 24.51 Gb clean data, after removing low-quality reads and mapping to the reference genome, 170,204,174 reads were retained. The number of reads per sample ranged from 7,938,124 to 12,884,908, with paired-end mapping rates above 87.53%. GC content ranged from 35.24% to 36.17%, Q30 values ranged from 90.64% to 92.91%, and mean coverage depth ranged from 20.07 to 28.41 (Table 2). A total of 791,621 SNPs were identified across the 19 cowpea accessions. The number of homozygous SNPs per accession ranged from 26,948 to 34,834, whereas heterozygous SNPs ranged from 8,641 to 21,439. Among them, jiang17 had the fewest homozygous SNPs, while 1858 had the largest numbers of both homozygous and heterozygous SNPs. The transition/transversion (Ti/Tv) ratio was approximately 2.1 across all accessions (Table 3).

**Fig 1:**
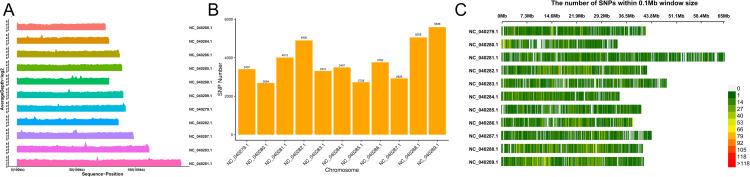
Mutation information statistics. A. Depth distribution of ddRAD-seq reads mapped to the cowpea genome. The left y-axis indicates read depth (plotted on log2 scale). The right y-axis shows chromosome names. The x-axis indicates chromosome length. B. Distribution of the number of raw SNP markers across chromosomes. C. Density distribution of raw SNP markers. The x-axis indicates chromosome length, the y-axis indicates chromosome names, and colors represent the number of SNPs within each window.

To further evaluate sequencing quality and the characteristics of SNPs, we analyzed sequencing depth distribution, SNP counts and densities based on ddRAD-seq data. The average sequencing depth across chromosomes showed an overall stable distribution without obvious depth depletion or excessive enrichment, indicating uniform genome coverage and sufficient depth for SNP discovery (Fig 1A). Significant differences were observed in the number of SNPs among chromosomes: chromosome NC_040289.1 contained the largest number of SNPs (5,588), whereas NC_040280.1 had the fewest SNPs (2,684) (Fig 1B). These differences reflect chromosome-level variation in genetic diversity and provide a basis for prioritizing chromosomes in subsequent marker development. Using a 0.2 Mb sliding window, SNP density analyses revealed heterogeneity among chromosomes. Chromosome NC_040281.1 maintained relatively high SNP density across its length, whereas some chromosomes exhibited local troughs of low SNP density. The color gradient (green to red representing low to high SNP counts) indicated that most windows contained 1–85 SNPs, and only a small number of windows showed highly variable regions with >169 SNPs, suggesting that cowpea genomic variation is broadly distributed but also contains localized hotspots (Fig 1C). Overall, the abundance and clear distribution patterns of SNPs provide a solid molecular foundation for KASP marker development, genetic diversity analyses, and DNA fingerprint construction.

**Table 2:** Statistics on whole-genome sequencing of 19 genotypes and assembly quantity statistics.

sample	Total Reads	Mapped Paired Reads	GC(%)	Q30 (%)	Average Cover Depth
170	8519330	8368674(98.23%)	35.72	92.81	21.11
17FY	9485818	8302490(87.53%)	35.92	92.45	20.07
18415	8766178	8634310(98.50%)	35.87	92.91	21.92
18442	8331358	8280208(99.39%)	35.24	92.79	25.8
18443	8514034	8413454(98.82%)	35.47	92.77	23.6
1858	8521658	8449674(99.16%)	35.3	90.64	22.32
19302-2-4	8154588	7742220(94.94%)	36.06	92.12	22.08
20351	9447530	8742348(92.54%)	35.8	92.78	23.26
20479	9492924	9083562(95.69%)	36.02	92.76	23.56
22565	9670652	9591158(99.18%)	35.56	92.71	25.18
22572	12884908	12801680(99.35%)	35.54	92.72	28.41
22599	8330818	7908320(94.93%)	35.79	92.25	22.8
JJ40-1-1	8282304	8172852(98.68%)	35.92	92.72	20.48
jiang12	8045084	7938958(98.68%)	36.17	92.48	21.57
jiang14	7938124	7703576(97.05%)	36.06	92.62	20.31
jiang17	8246860	8183850(99.24%)	35.34	92.66	21.33
jiang30	9414600	9251762(98.27%)	35.66	92.77	22.77
jiang6	9579768	9430600(98.44%)	35.7	92.84	24.69
p-3	8577638	8515410(99.27%)	36.05	92.76	23.29

**Table 3:** Statistical table of original variation information.

Sample	SNP number	Transition	Transversion	Homozygosity	Heterozygosity	Ti/Tv
170	46072	31419	14653	34443	11629	2.144202552
17FY	44947	30348	14599	34035	10912	2.078772519
18415	47771	32860	14911	37651	10120	2.203742204
18442	36724	25082	11642	27760	8964	2.154440818
18443	37184	25192	11992	28543	8641	2.100733823
1858	56273	38022	18251	34834	21439	2.083283108
19302-2-4	39434	26854	12580	29125	10309	2.134658188
20351	41892	28396	13496	31548	10344	2.104030824
20479	44893	30637	14256	33619	11274	2.149060045
22565	42962	29352	13610	32299	10663	2.156649522
22572	38394	25899	12495	29234	9160	2.0727491
22599	37696	25560	12136	27950	9746	2.106130521
JJ40-1-1	41385	28122	13263	30747	10638	2.120334766
jiang12	36942	25063	11879	27349	9593	2.109857732
jiang14	41253	28080	13173	30401	10852	2.131632885
jiang17	36501	24683	11818	26948	9553	2.088593671
jiang30	41526	28073	13453	31048	10478	2.086746451
jiang6	41001	28017	12984	30333	10668	2.157809612
p-3	38771	26485	12286	28433	10338	2.155705681

### 3.2 Genetic diversity evaluation of cowpea varieties using SNP markers from ddRAD-Seq

**Fig 2:**
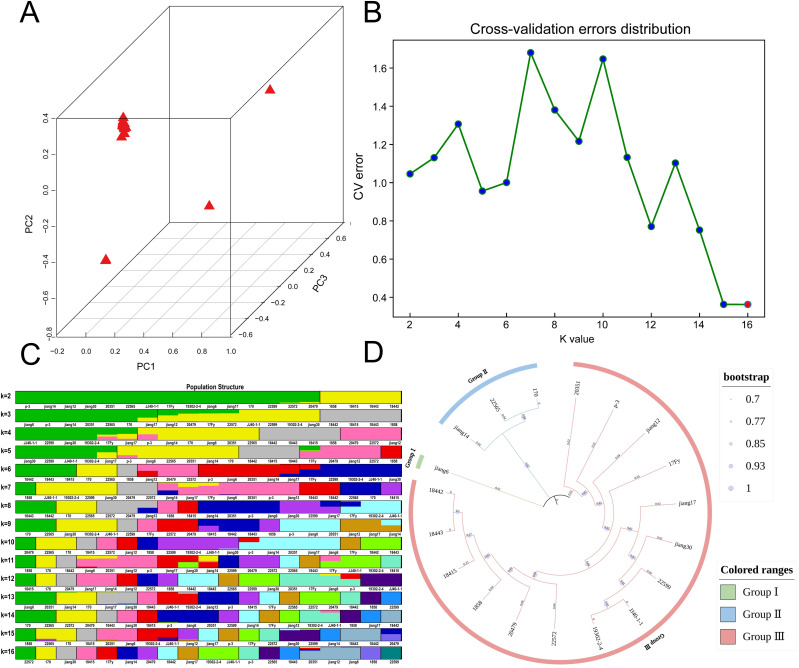
Population genetic analyses of 19 cowpea accessions based on polymorphic SNPs. (A) Principal component analysis (PCA). (B) Cross-validation error under different K values. (C) Population structure inferred by ADMIXTURE at different K values. (D) Phylogenetic tree constructed using the maximum-likelihood (ML) method implemented in FastTree v2.1.9. Branch numbers indicate support values.

Principal component analysis (PCA) based on SNPs was performed for the 19 cowpea germplasm. The variance explained by principal components PC1, PC2, and PC3 was 31.06%, 22.67%, and 12.62%, respectively, cumulatively explaining 66.35% of the total genetic variation, indicating that the first three PCs captured the major genetic differences among accessions. In the two-dimensional scatter plot of PC1 and PC2, the 19 accessions were broadly dispersed without forming clearly separated clusters, suggesting a certain degree of genetic differentiation among accessions. Several accessions showed a wide spread along PC1 but were relatively concentrated along PC2, indicating that PC1 contributed most to distinguishing the accessions. In the three-dimensional distribution including PC3, most accessions showed PC3 values from −0.4 to 0.4, further reflecting differences at specific loci (Fig 2A). Overall, the PCA pattern may be influenced by domestication history, gene flow, and artificial selection, and provides important information for subsequent diversity analyses and core germplasm selection.

Population structure analysis based on ddRAD-seq data indicated that the cross-validation (CV) error reached its minimum at K = 15 (Fig 2B). The inferred genetic structure of the tested cowpea accessions became progressively more differentiated as the number of assumed ancestral populations (K) increased (Fig 2C). At K = 2, the accessions could be divided into two major genetic components (green and yellow), with most accessions dominated by a single component and only a few showing limited admixture. At K = 3, an additional gray component emerged and the yellow component began to exhibit substructure. As K increased from 4 to 16, additional components appeared sequentially (pink, red, blue, purple, etc.), and the genetic backgrounds of the accessions gradually displayed complex mosaic patterns composed of multiple components. Overall, the differentiation was relatively clear at low K values (K = 2–4), reflecting basic genetic groupings, whereas finer differentiation at higher K values (K ≥ 5) revealed potential substructure and gene flow events. These results provide important information on genetic structure for defining cowpea genetic groups, mining genetic resources, and constructing breeding populations.

A phylogenetic tree constructed from the ddRAD-seq data using the maximum-likelihood (ML) method illustrated the evolutionary relationships and clustering patterns among the 19 cowpea accessions (Fig 2D). Based on the tree topology, the accessions were divided into three major clades. Group I was centered on jiang6 and clustered with 20351 and p-3. Group II included jiang14, 170, and 22565, which clustered closely together. Group III contained the largest number of accessions, including jiang17, jiang30, 22599, 19302-2-4, JJ40-1-1, 18415, 18442, and 18443. Support values for pairs such as 18442/18443 and jiang30/22599 were high, indicating similar genetic backgrounds and stable clustering. Overall, closely related accessions tended to cluster together on the tree. This clustering pattern was generally consistent with the diversity analyses and supported the usefulness of ddRAD-seq for evaluating genetic relationships among cowpea accessions.

### 3.3 Design of KASP primers and selection of Core SNP sites

**Fig 3:**
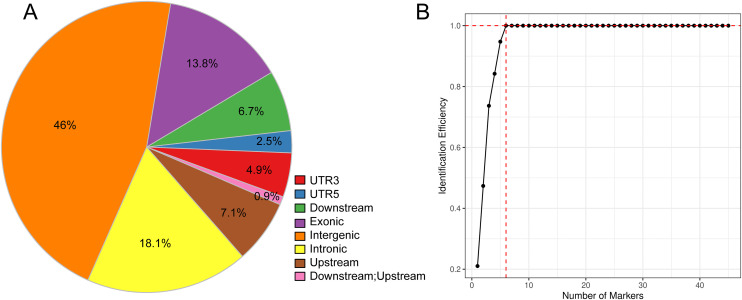
Distribution of SNP markers and marker identification efficiency.

Primer design was performed for the 648 screened SNP markers, resulting in 326 SNPs primer were designed, which were subsequently converted into KASP markers. For these 326 SNPs, PIC values ranged from 0.355 to 0.375, and observed heterozygosity ranged from 0 to 1 (S2 Table). These markers were distributed across functional categories, including 3′ UTR, 5′ UTR, downstream, exonic, intergenic, intronic, upstream, and upstream/downstream regions (Fig 3A). To evaluate identification efficiency using the 45 exonic-region markers, the identification efficiency increased rapidly from ~0.20 to 1.00 when the number of markers increased from 0 to 6; when the marker number reached 6 or more, the identification efficiency remained at the saturated level of 1.00 without substantial fluctuation across the full set of 45 markers (Fig 3B). These results indicate that only six core KASP markers from exonic regions are sufficient for accurate identification.

### 3.4 Construction of DNA fingerprints

**Fig 4:**
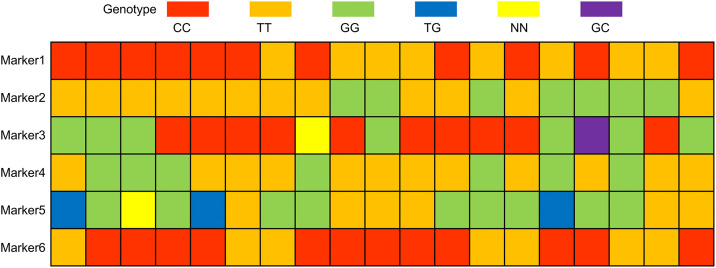
Marker-based fingerprint map. Each row represents one marker and each column represents one sample. Homozygous genotypes (C/C, T/T, G/G) are indicated by red, orange, and green colors, respectively; heterozygous genotypes are indicated by blue and purple; missing genotypes are indicated by yellow.

A fingerprint map constructed based on the six KASP markers (Marker1-Marker6) visually presents the distribution of genotype polymorphisms among the tested cowpea accessions (Fig 4). The DNA fingerprints showed that the six KASP markers could discriminate the 19 accessionsBased on the SNP genotypes of each accession, each sample was assigned a specific QR code (S1 Fig).

From the fingerprint characteristics, the genotypes of each marker differed clearly among accessions. Marker1 and Marker6 were mainly represented by red (homozygous genotype) and orange (homozygous genotype). Marker2 and Marker4 were mainly green (homozygous genotype) and orange (homozygous genotype), forming relatively stable genotype groupings across accessions. Marker3 was red (homozygous genotype) and green (homozygous genotype) in most accessions, but purple (heterozygous genotype) and yellow (missing genotype) were also observed, indicating genetic variation or missing data for this marker in some accessions. Marker5 also displayed its own distinctive color pattern and discriminative genetic features, with genotype differences observed in some accessions. Overall, the combination of genotypes across the six markers generated unique fingerprint features for the tested accessions, allowing most samples to be distinguished. Only a few accessions (e.g., 18442 and 18443) showed genotype overlap at some markers and may require additional core markers for further discrimination. These results indicate that the selected KASP markers are polymorphic and can serve as effective tools for molecular identification of cowpea accessions, supporting further improvement of fingerprinting systems and accurate germplasm identification.

**Fig 5:**
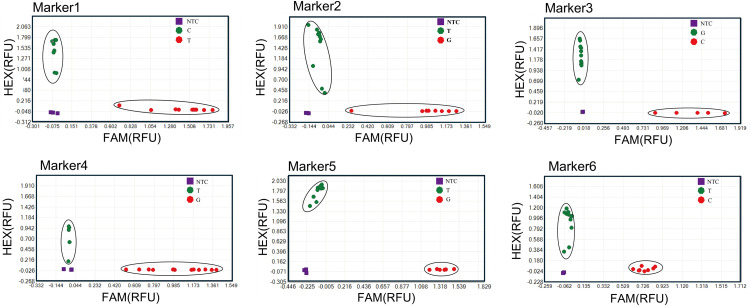
Allelic genotyping of 19 cowpea accessions using six KASP marker assays. Green circles and red circles represent the homozygous genotypes; and the purple squares on the bottom left of the plot are no-template controls.

To further validate the effectiveness of the fingerprinting profile, KASP markers were developed from six core SNP loci, and genotyping was conducted for 19 cowpea accessions. The results showed that all six markers produced successful genotype calls. Only homozygous genotypes were observed across the 19 accessions; green and red circles represent two different homozygous genotypes. Marker 1 carried allele C or T; Marker 2 carried allele T or G; Marker 3 carried allele G or C; Markers 4 and 5 carried allele T or G; and Marker 6 carried allele T or C. Overall, based on the genotyping results, these six KASP markers could discriminate the 19 cowpea accessions (Fig 5). There results indicate that the selected KASP markers are polymorphic and can serve as effective tools for molecular identification of cowpea materials, providing support for further improvement of the fingerprinting system and accurate identification of accessions.

## 4. Discussion

Cowpea is a globally important legume crop, yet its genetic improvement and variety protection have been constrained by the lack of efficient molecular markers and insufficient population genetic data. As a core reduced-representation sequencing approach, ddRAD-seq is a high-throughput genotyping method based on restriction sites across the whole genome. It sequences DNA fragments generated after complete restriction digestion and uses two restriction endonucleases to generate fragments of different sizes, thereby increasing the number of genotyped loci [23]. This approach can generate reliable results with relatively low sequencing output, reduce potential DNA loss during library construction, and effectively decrease genome complexity, thereby simplifying library preparation [24]. Therefore, ddRAD-seq has been widely applied in phylogenetics and species identification [25]. In this study, ddRAD-seq was applied to 19 cowpea germplasm accessions, yielding 24.51 Gb of clean data and, after filtering, 170,204,174 reads. Reads per sample ranged from 7,938,124–12,884,908; Q30 values were 90.64%–92.91%; GC content was 35.24%–36.17%; and mean sequencing depth was 20.07–28.41. Reads were aligned to the reference genome with a mapping rate exceeding 87.53%. Overall, the sequencing data met high-quality standards and provided a solid basis for subsequent SNP discovery.

Many studies have characterized cowpea germplasm using diverse molecular marker systems; in the present study, we used SNP markers for variety identification. Compared with conventional marker types, SNP markers offer high polymorphism, codominant inheritance, high density, and high throughput, and are widely regarded as ideal DNA markers [26]. Across the 19 core cowpea accessions, a total of 791,621 SNP loci were identified. These SNPs were broadly distributed across the 11 chromosomes, with localized hotspots of high variation. For example, chromosome NC_040289.1 contained 5,588 SNPs, whereas NC_040280.1 contained only 2,684 SNPs; this distribution pattern provides a basis for selecting uniformly distributed core markers in subsequent work (Fig 1B). In addition, the transition/transversion (Ti/Tv) ratio was approximately 2.1, consistent with variation patterns in plant genomes and supporting the authenticity of the SNP calls (Table 3).

Developing broadly applicable SNP markers requires substantial background divergence among the tested materials. Based on the SNP loci obtained by sequencing, we performed principal component analysis (PCA) and phylogenetic analysis for the 19 cowpea accessions. The PCA results showed a relatively dispersed distribution of the 19 accessions, indicating a certain degree of genetic differentiation among them (Fig 2A). Phylogenetic analysis further suggested that the 19 accessions could be divided into three clades (Fig 2D). Together, these results indicate appreciable diversity among the accessions, which is favorable for identifying core SNP loci.

Because SNP genotyping often requires complex detection technologies and can be costly, a substantial gap remains between SNP development and practical application in breeding [27]. To date, KASP genotyping based on SNPs has been successfully applied in rice, wheat, Chinese cabbage, and other species [28–30]. KASP substantially reduces genotyping costs while improving practicality, and thus serves as a powerful tool for rapid germplasm identification and marker-assisted breeding [31]. In this study, 648 loci meeting the screening criteria were progressively selected from 791,621 SNP loci, of which 326 were successfully converted into KASP markers, achieving a conversion efficiency of 50.3%. The polymorphism information content (PIC) values ranged from 0.355 to 0.375. The relatively narrow PIC range of the developed KASP markers is likely attributable to the marker selection strategy used. Because candidate SNPs were progressively filtered for sequencing quality, polymorphism, unique genomic mapping, and KASP conversion suitability, and only loci with PIC values > 0.35 were retained, the final set of markers showed a relatively concentrated PIC distribution. Functional region annotation showed that the 326 KASP markers covered eight categories, including 3′ UTR, exons, introns, and intergenic regions; exonic markers accounted for 13.8% (45 markers) (Fig 3A). Such markers are more likely to be associated with phenotypic traits and provide resources for developing functional markers. Marker-number evaluation indicated that when the marker set reached six or more, identification efficiency remained at the saturated level of 1.00 (Fig 3B). After converting six SNP loci into KASP markers, genotyping demonstrated that these six core KASP markers enabled accurate identification of the 19 cowpea accessions and supported the construction of a fingerprinting profile (Fig 4). These results confirm the feasibility and reliability of developing molecular markers using ddRAD-seq, laying a solid technical foundation for large-scale, high-throughput DNA fingerprinting of additional cowpea varieties and providing technical support for variety authentication, seed authenticity testing, and intellectual property protection in cowpea. In practical marker-assisted breeding programs, this KASP marker panel could provide a rapid and cost-effective means of verifying parental identity, confirming the genetic purity of breeding materials, and, once the markers are linked to target agronomic traits, tracking informative alleles to support early-generation selection and more efficient parental combination design.Nevertheless, limitations remain. Only 19 cowpea accessions were included. Although core inbred lines with distinct geographic origins and agronomic traits were selected, the relatively limited sample size may affect the comprehensiveness and representativeness of the genetic diversity analyses. Future studies should expand germplasm sampling to include more accessions with diverse geographic origins and genetic backgrounds to more comprehensively elucidate cowpea genetic diversity and broaden the applicability of the fingerprinting system.

## 5. Conclusion

In this study, 19 cowpea accessions were subjected to high-throughput ddRAD-seq. After strict quality control, 13,469 high-quality SNPs were obtained. Through multi-criteria filtering, 326 KASP molecular markers were successfully developed; these markers were evenly distributed across the cowpea genome and exhibited good stability and polymorphism. Six core KASP markers were further selected, and their genotyping results were accurate and reliable. Only homozygous genotypes were obserbed at these markers in the tested accessions, with clear genotype differentiation that enabled effective discrimination of cowpea germplasm. Using the six core KASP markers, a DNA fingerprinting profile of the 19 accessions was successfully constructed, which distinguished most tested materials and can be applied in cowpea germplasm identification and marker-assisted breeding. Future work should integrate additional cowpea accessions and develop functional KASP markers tightly linked to key agronomic traits to futher improve the fingerprinting system and provide more comprehensive technical support for cowpea genetic improvement and sustainable industry development.

## Supporting information

S1 Fig Nineteen cowpea accessions with two-dimensional barcodes. The two-dimensional barcodes were generated using an online platform (https://www.hlcode.cn/?p=bdmcp). Permission for use in this publication was obtained from the provider.
(TIF)

S1 Table Primer sequences of KASP-specific primers. (DOCX)

S2 Table Information of 326 SNPs. (XLSX)
