# Supplementary material for: Development of KASP molecular markers and construction of fingerprinting for cowpea (Vigna unguiculata (L.) Walp.) based on ddRAD-Seq
Source: PLoS One. 2026 Aug 14;21(8):e0343756. doi: 10.1371/journal.pone.0343756 (PMC13475943; doi:10.1371/journal.pone.0343756)
Supplement: Supplementary file 1 [file pone.0343756.s002.docx]

Table S1 Sequences of KASP-specific primers used in this study

| **ID** | **Allele-specific primer 1** | **Allele-specific primer 2** | **Common primer** |
| --- | --- | --- | --- |
| Marker1 | GAAGGTGACCAAGTTCATGCTGAGTATCTGACACGGCATCATTTTC | GAAGGTCGGAGTCAACGGATTGAGTATCTGACACGGCATCATTTTT | GACGAGTTCAGGCATCTGCTTTCATTAAG |
| Marker2 | GAAGGTGACCAAGTTCATGCTAACGTGTCAATGAAACCAAGTGCAT | GAAGGTCGGAGTCAACGGATTAACGTGTCAATGAAACCAAGTGCAG | TAACCAAAATATGTTGTCTGAAGGACGGG |
| Marker3 | GAAGGTGACCAAGTTCATGCTTTTCTTCTTCCTCCTGATAGAGAGG | GAAGGTCGGAGTCAACGGATTTTTCTTCTTCCTCCTGATAGAGAGC | ACAGAGCCAATGGTCGTCTCACAATACAT |
| Marker4 | GAAGGTGACCAAGTTCATGCTAGCTAGAAGCTTCCCGTGGTGATAT | GAAGGTCGGAGTCAACGGATTAGCTAGAAGCTTCCCGTGGTGATAG | ATATTCCAATTTCTACCAGCCAATACTGC |
| Marker5 | GAAGGTGACCAAGTTCATGCTAATGCCATATTCCAAACTAATAGAT | GAAGGTCGGAGTCAACGGATTAATGCCATATTCCAAACTAATAGAG | AGCAACCTTGGCCAACATGACTGATAAGC |
| Marker6 | GAAGGTGACCAAGTTCATGCTCAGAGCTCAACTTAGTGAACGCCCT | GAAGGTCGGAGTCAACGGATTCAGAGCTCAACTTAGTGAACGCCCC | TAAGCTAATGCAATTGCTTGGTTGAATAC |
